# Manual aspiration of a pneumothorax after CT-guided lung biopsy: outcomes and risk factors

**DOI:** 10.1259/bjr.20220366

**Published:** 2023-06-28

**Authors:** Michael Vinchill Chan, Zahra Afraz, Ya Ruth Huo, Sonja Kandel, Patrik Rogalla

**Affiliations:** 1 Department of Medical Imaging, University Health Network, University of Toronto, Toronto, Canada; 2 Department of Radiology, Concord Repatriation General Hospital, NSW, Concord, NSW, Australia; 3 Concord Hospital Clinical School, University of Sydney, NSW, Concord, Australia

## Abstract

**Objective:**

Quantify the outcomes following pneumothorax aspiration and influence upon chest drain insertion.

**Methods:**

This was a retrospective cohort study of patients who underwent aspiration for the treatment of a pneumothorax following a CT percutaneous transthoracic lung biopsy (CT-PTLB) from January 1, 2010 to October 1, 2020 at a tertiary center. Patient, lesion and procedural factors associated with chest drain insertion were assessed with univariate and multivariate analyses.

**Results:**

A total of 102 patients underwent aspiration for a pneumothorax following CT-PTLB. Overall, 81 patients (79.4%) had a successful pneumothorax aspiration and were discharged home on the same day. In 21 patients (20.6%), the pneumothorax continued to increase post-aspiration and required chest drain insertion with hospital admission. Significant risk factors requiring chest drain insertion included upper/middle lobe biopsy location [odds ratio (OR) 6.46; 95% CI 1.77–23.65, *p* = 0.003], supine biopsy position (OR 7.06; 95% CI 2.24–22.21, *p* < 0.001), emphysema (OR 3.13; 95% CI 1.10–8.87, *p* = 0.028), greater needle depth ≥2 cm (OR 4.00; 95% CI 1.44–11.07, *p* = 0.005) and a larger pneumothorax (axial depth ≥3 cm) (OR 16.00; 95% CI 4.76–53.83, *p* < 0.001). On multivariate analysis, larger pneumothorax size and supine position during biopsy remained significant for chest drain insertion. Aspiration of a larger pneumothorax (radial depths ≥3 cm and ≥4 cm) had a 50% rate of success. Aspiration of a smaller pneumothorax (radial depth 2–3 cm and <2 cm) had an 82.6% and 100% rate of success, respectively.

**Conclusion:**

Aspiration of pneumothorax after CT-PTLB can help reduce chest drain insertion in approximately 50% of patients with larger pneumothoraces and even more so with smaller pneumothoraces (>80%).

**Advances in knowledge:**

Aspiration of pneumothoraces up to 3 cm was often associated with avoiding chest drain insertion and allowing for earlier discharge.

## Introduction

CT-guided percutaneous transthoracic lung biopsy (CT-PTLB) is a minimally invasive procedure for acquiring lung tissue. A pneumothorax is the most common complication with an incidence ranging from 18.8% to 45% and a chest drain insertion rate ranging from 3.1% to 20%.^1–5^ In certain institutions, aspiration of a pneumothorax is often performed with the aim of treating the pneumothorax and avoiding chest drain insertion. The most common criteria for attempting pneumothorax aspiration is often dependent on the pneumothorax size, however, the criteria vary between institutions and proceduralists.

Despite pneumothorax aspiration being commonly performed in certain institutions, there is very little published literature quantifying which patients avoid a chest drain insertion and which patients require a chest drain despite aspiration. The largest published study in 112 patients demonstrated aspiration of a smaller pneumothorax (<543 ml aspirated air) was significantly associated with no chest drain insertion compared to those who had a larger pneumothorax (>543 ml) (94% vs  49% not requiring a chest drain).^6^ This study highlighted that manual aspiration of a pneumothorax is an effective technique to reduce chest drain insertion, even for a large pneumothorax. There are only a few other studies quantifying the outcomes of aspiration following a pneumothorax in individual case reports, case series or incidentally recorded as part of a larger study of pneumothorax management.^7–10^ The lack of published research into this procedure makes it difficult for a proceduralist to identify which patient would benefit from pneumothorax aspiration versus immediate chest drain insertion.

The aim of this study is to assess and quantify risk factors for chest drain insertion following manual aspiration, such as pneumothorax size, patient characteristics, lesion factors and procedural factors.

## Methods

This is a retrospective cohort study which included all patients who underwent manual aspiration for the treatment of pneumothorax after CT-PTLB core-biopsy from January 1, 2010 to October 1, 2020 at Toroto General Hospital. Institutional ethics committee approval was attained. Exclusion criteria included fine needle aspiration (FNA), non-lung biopsy (*i.e.* pleural or mediastinal), chest drain insertion without aspiration attempt or intentional capnothorax was created for biopsy.

Medical records, procedural images, and pathology results were reviewed by two investigators (MVC and ZA). Patient demographics, comorbidities, lesion characteristics, and operator technique were extracted.

**Figure 1.**
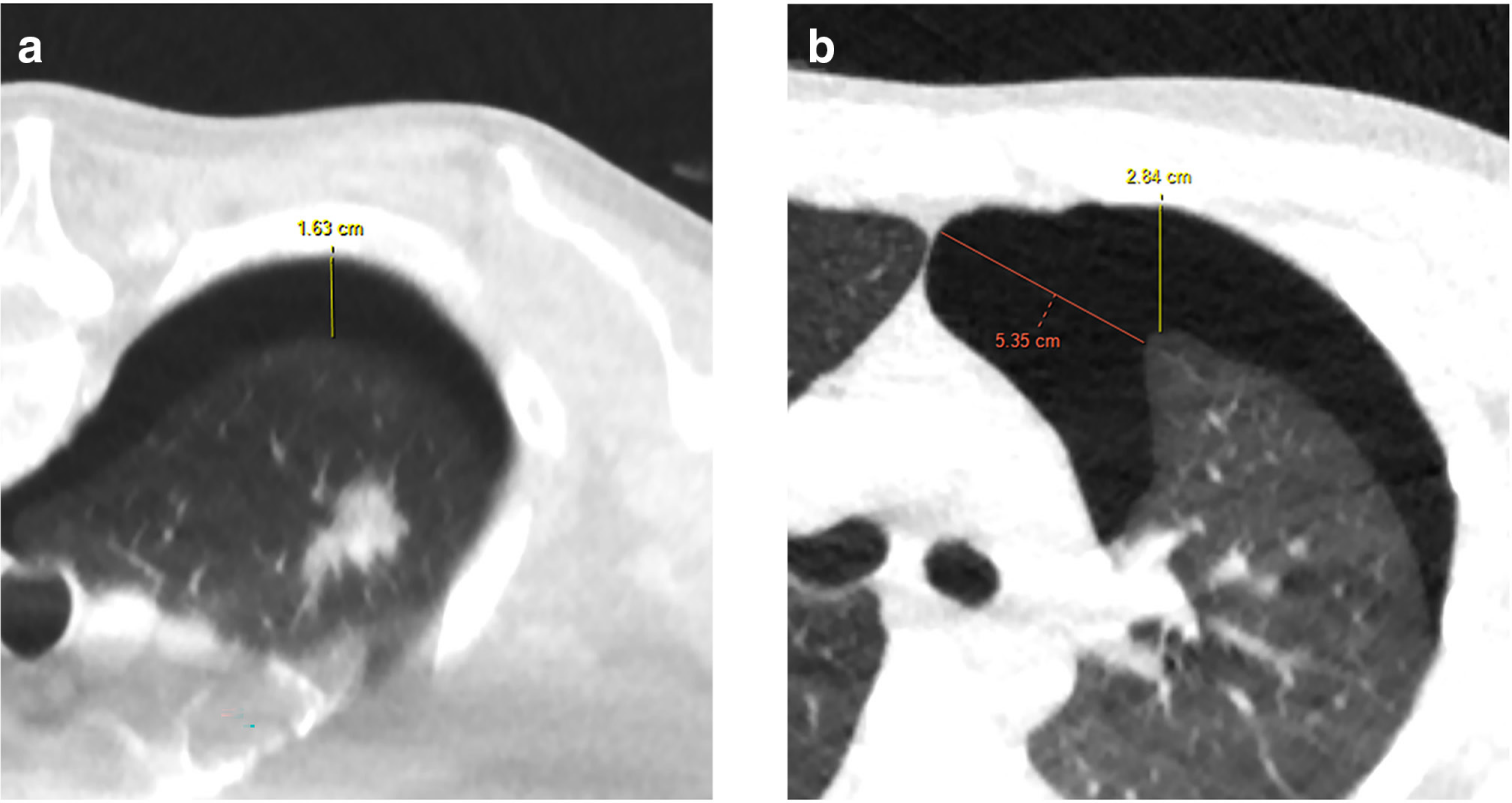
Examples of how the longest radial depth was measured. (a) Greatest radial distance in prone positioning. (b) Greatest radial depth (vertical anterior-posterior line) was taken instead of the larger oblique distance (more medial oblique line) on supine position.

The longest perpendicular distance on axial imaging of the pneumothorax from the lung surface (visceral pleura) to the parietal pleura was measured (Figure 1). This measuring technique was chosen to maintain consistency between the different positions of patients.

### CT-guided lung biopsy technique

All lung biopsies were performed in an Aquilion One Vision CT scanner (Canon Medical Systems, Tochigi, JP) by thoracic imaging fellows and thoracic radiology attendings. Biopsy techniques included both CT-fluoroscopy and helical CT guidance; coaxial and non-coaxial techniques and needle gauges varying from 17- to 19-Gauge. The non-coaxial technique utilized an 18-Gauge Biopince device (Argon Medical Devices, TX). Coaxial approach was performed with an 18- or 20-Gauge Temno needle (Merit Medical, UT) with a corresponding 17- or 19-Gauge coaxial needle. No tract sealant was utilized in coaxial technique lung biopsies in this cohort. Patient positioning was dependent on operator preference. Patients were placed in a supine position following biopsy. Procedure time was taken from the timestamp from the biopsy needle first imaged in the skin to the final image. The presence of emphysema was based on a region of interest measurement of −860 HU along the expected needle tract, concordant to reported values of emphysema in expiration.^11^

### Manual aspiration technique

The decision to aspirate the pneumothorax was made by the proceduralist performing the lung biopsy. Reasons included enlarging pneumothorax during the procedure or if the pneumothorax size appeared significant on visual assessment to the proceduralist by the conclusion of the procedure. Consideration for manual aspiration after conclusion of the procedure included respiratory-related clinical deterioration with known pneumothorax confirmed by CT and progressively enlarging pneumothorax on serial routine chest X-rays after the procedure.

For proceduralists who used a coaxial technique (75.5%), aspiration was performed through the coaxial guide needle. For proceduralists who used a non-coaxial technique (24.5%), or delayed pneumothorax, a separate 10-Fr catheter was placed through the anterior chest wall with the patient supine with the cannula directed superiorly and laterally. A 3-way valve was attached to the coaxial needle hub or inserted catheter before manual aspiration or wall suction.

Re-imaging using CT-fluoroscopy or helical CT was performed at the time of aspiration to ensure decreasing pneumothorax size. Serial chest X-rays 1- and 2 hour post-aspiration were performed to ensure stability prior to discharge. If there was confirmed failure of aspiration, a drain was placed. For stable patients, mid-procedure with coaxial technique, this was converted to an 8-Fr drain via Seldinger technique. For delayed presentations, unstable patients and non-coaxial technique, if attempts to aspirate via a 10-Fr catheter were not successful, these would be converted to have the drain left in. All patients with a drain were eventually connected to underwater seal drainage and admitted for observation and management.

### Statistical analysis

Statistical analysis was performed with SPSS for Windows, v. 24.0 (SPSS, Munich, Germany). Univariate analysis was performed using Student’s *t*-test, χ^2^ test and Fisher’s exact test to identify factors associated with an increased risk of chest drain insertion following aspiration for the treatment of a pneumothorax. Clinically significant variables and variables with a *p*-value of <0.10 were included in the multivariate analysis. A *p*-value of <0.05 was considered statistically significant.

## Results

**Figure 2.**
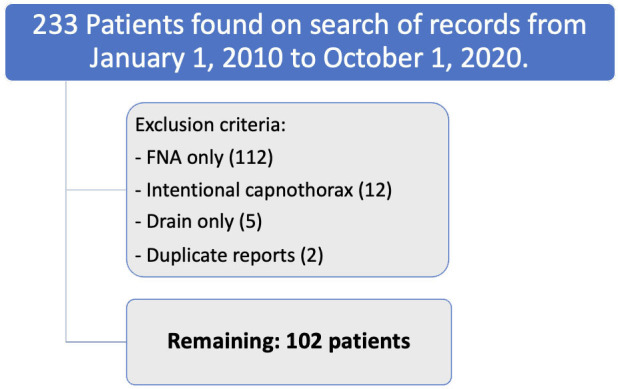
Eligibility criteria for inclusion and reasons for exclusion. FNA, fine needle aspiration.

From January 1, 2010, to October 1, 2020, 233 potentially eligible studies were obtained. Excluded studies included 112 due to FNA, 12 due to intentional capnothorax, 5 due to chest drain insertion without aspiration and 2 duplicate reports. A total of 102 patients underwent aspiration for the treatment of a pneumothorax following CT-PTLB (Figure 2). Pneumothorax was detected on CT during or immediately after the biopsy in 87.3% and on the 1 hour post-biopsy CXR in 12.7%, where it had developed compared to final CT biopsy images (no pneumothorax seen during the biopsy on CT). The median age was 65.4 years (range 25–85). Conscious sedation was used in 80.4%. Emphysema was present in 50%. Mean lung lesion size was 28.8 mm (range 8–150 mm), with 55.9% in the upper/middle lobes and 44.1% in the lower lobes. The pulmonary lesions were solid in 89%, subsolid or ground glass in 10.7% and cavitation was seen in 8.8%. The mean needle depth was 23.8 mm (range 0–53 mm). There was more than one pleural puncture in 21.6%. Procedure time ranged from 4 to 72 min. Patient, lesion and procedural characteristics are summarized in Table 1.

**Table 1.** Baseline patient, lesion and procedural characteristics.

*Patient Characteristics*	
Sex, Female n (%)	35 (34.3%)
Age, mean years (range)	65.4 years (range 25–85)
Emphysema, n (%)	51 (50%)
Prior Chemotherapy, n (%)	22 (21.6%)
Prior Radiotherapy to thorax, n (%)	28 (27.5%)
Recent anticoagulation, n (%)	24 (23.5%)
***Lung Lesion Characteristics***	
Location	
Upper or middle lobe, n (%)	57 (55.9%)
Lower lobe, n (%)	45 (44.1%)
Fissure	
Abutting fissure, n (%)	32 (31.4%)
Not abutting fissure, n (%)	70 (68.6%)
Lesion size, mean mm (range)	28.8 mm (range 8–150)
***Biopsy Needle/Technique Characteristics***	
Biopsy Needle Crossed Fissure, n (%)	7 (6.9%)
CT scan technique	
Helical CT, n (%)	41 (40.2%)
CT Fluoroscopy, n (%)	61 (59.8%)
Biopsy Needle size	
>18G, n (%)	57 (55.9%)
≤18G, n (%)	45 (44.1%)
Biopsy device	
Biopince device, n (%)	24 (23.5%)
Temno device, n (%)	78 (76.5%)
Biopsy Technique	
Non-coaxial, n (%)	25 (24.5%)
Coaxial technique, n (%)	77 (75.5%)

Baseline patient, lesion and procedural characteristics (*n* = 102 patients).

### Pneumothorax aspiration success and chest drain insertion rates

Overall, 79.4% had a successful pneumothorax aspiration and were discharged home on the day of the procedure. The pneumothorax continued to increase in size despite aspiration in 20.6% and therefore a chest drain was inserted.

### Risk factors associated with chest drain insertion following pneumothorax aspiration

### Pneumothorax size (Longest perpendicular depth)

Chest drain insertion rates following pneumothorax aspiration was significantly higher for a larger pneumothorax compared to smaller pneumothorax, with a mean of 40.6 mm in radial depth for those that required chest drain insertion and 20.8 mm for those successfully treated with aspiration (*p* = 0.001) (Table 2). The chest drain insertion rate was 50% for a pneumothorax with the longest radial depth ≥4 cm, 50% for ≥3 cm, 36.8% for ≥2 cm and 0% for <2 cm (Table 2). Chest drain insertion rates was almost 16 times more likely if the pneumothorax longest radial depth was ≥3 cm compared to <3 cm (OR 16.00, 95% CI 4.76–53.83, *p* < 0.001). On subgroup analysis when removing small pneumothoraces under 1 cm, chest drain rate insertion for pneumothorax longest radial depth of >3 cm compared to 1–3 cm was similar (OR 13.00 95% CI 3.81–44.00). The univariate analyses for different pneumothorax radial depth cut-offs is summarized in Table 2 and Supplementary Table 1.

Supplementary Table 1.

STROBE Statement.

**Table 2.** Chest drain insertion rates following pneumothorax aspiration for patient, lung

	Chest drain insertion after peumothorax aspiration		OR (95% CI)
	Group A	Group B	
**PATIENT CHARACTERISTICS**			
Sex: Female [A] *vs* Male [B]	12.5%	25.8%	2.44 (0.81–7.29)
Age: <70y [A] *vs* ≥70y [B]	20.7%	20.5%	0.99 (0.37–2.60)
Conscious sedation: None [A] *vs* Yes [B]	15.8%	20.7%	1.40 (0.36–5.34)
Emphysema: No [A] *vs* Yes [B]	11.8%	29.4%	3.13 (1.10–8.87)^*^
Recent anticoagulants: No [A] *vs* Yes [B]	20.5%	20.8%	1.02 (0.33–3.15)
Prior Chemotherapy: No [A] *vs* Yes [B]	21.3%	21.6%	0.82 (0.25–2.76)
Prior Radiotherapy: No [A] *vs* Yes [B]	25.7%	7.1%	0.22 (0.05–1.03)
**LUNG LESION CHARACTERISTICS**			
Location: Upper or middle lobe [A] *vs* lower [B]	31.6%	6.7%	6.46 (1.77–23.65)^***^
Abuts fissure: No [A] *vs* Yes [B]	21.4%	18.8%	0.85 (0.29–2.43)
Lesion type (subsolid [A]/ solid [B])	9.1%	22.0%	2.82 (0.34–23.34)
Lesion type: cavitation (No [A]/ Yes [B])	22.6%	0%	0.89 (0.823–0.96)
**BIOPSY NEEDLE CHARACTERISTICS**			
Needle technique (Non-coaxial [A]/ Coaxial [B])	28%	18.2%	0.57 (0.20–1.62)
Needle type (Biopince [A]/ Temno [B])	29.2%	17.9%	0.53 (0.19–1.52)
Needle gauge (>18G (small) [A]/ ≤18G (big) [B])	17.5%	24.4%	1.52 (0.58–3.99)
**BIOPSY TECHNIQUE**			
CT technique (Helical [A]/ Fluoroscopy [B]	17.1%	23%	1.45 (0.53–3.97)
Crosses fissure: No [A] *vs* Yes [B]	21.1%	14.3%	0.625 (0.07–5.49)
Pleural punctures: 1 [A] *vs* >1 [B]	23.8%	9.1%	0.32 (0.07–1.50)
Position: Prone [A] *vs* Supine [B]	8.1%	38.2%	7.06 (2.24–22.21)^***^
Needle depth:			
<1 cm [A] *vs* ≥1 cm [B]	7.7%	25.0%	4.00 (0.86–18.53)
<2 cm [A] *vs* ≥2 cm [B]	11.5%	34.7%	4.00 (1.44–11.07)^**^
<3 cm [A] *vs* ≥3 cm [B]	13.9%	36.7%	3.59 (1.32–9.75)^**^
<4 cm [A] *vs* ≥4 cm [B]	15.4%	37.5%	3.30 (1.18–9.25)^*^
<5 cm [A] *vs* ≥5 cm [B]	16.7%	50.0%	5.00 (1.41–17.63)^**^
Longest radial pneumothorax depth			
< 2 cm [A] *vs* ≥2 cm [B]	0%	36.8%	N/A
< 3 cm [A] *vs* ≥3 cm [B]	6.3%	50.0%	16.00 (4.76–53.83)^***^
< 4 cm [A] *vs* ≥4 cm [B]	11.5%	50.0%	7.67 (2.66–22.12)^***^
Diagnostic success: No [A] *vs* Yes [B]	9.7%	25.4%	3.17 (0.86–11.69)
Procedure time: ≤30 min [A] *vs* >30 min [B]	18.0%	24.4%	1.44 (0.56–3.86)

95%CI - 95%, 95% Confidence Interval; Green Shading, utilized for regression analysis; N/A, Not Applicable;OR, odds ratio.

* *p*-value <0.05

** *p*-value <0.01

*** *p*-value <0.001

On multivariate analysis, a larger pneumothorax size remained significantly associated with chest drain insertion following aspiration (≥3 cm *vs* <3 cm, adjusted OR 5.87, 95% CI 1.47–23.43, *p* = 0.012).

### Supine position

Chest drain insertion rates following pneumothorax aspiration was significantly higher in patients who were biopsied in the supine position (38.2%) compared the prone position (8.1%) on univariate analysis (OR 7.06, 95% CI 2.24–22.21, *p* < 0.001, Table 2) as well as multivariate analysis (adjusted OR 5.99, 95% CI 1.11–35.32, *p* = 0.048) (Table 3).

**Table 3.** Multivariate analysis using binary logistic regression for chest drain insertion after pneumothorax aspiration

Characteristic	Adjusted OR	(95% CI)	*p*-value
Longest radial PTX depth ≥3 cm (*vs* <3 cm)	5.87	(1.47–23.43)	***0.012***
Supine biopsy position ( *vs* Prone)	5.99	(1.11–35.32)	***0.048***
Upper/middle lobe ( *vs* lower lobe)	1.81	(0.22–15.00)	*0.584*
Emphysema ( *vs* no emphysema)	2.51	(0.58–10.90)	*0.219*
Needle distance ≥2 cm (*vs* <2 cm)	3.75	(0.86–16.38)	*0.079*
Prior adiotherapy ( *vs* no radiotherapy)	0.39	(0.06–2.46)	*0.315*

CI, confidence interval; OR, odds ratio; PTX, pneumothorax.

### Upper/Middle lobe location

Chest drain insertion rates following pneumothorax aspiration was significantly higher following biopsy of lesions in the upper or middle lobes (31.6%) compared to the lower lobes on univariate analysis (OR 6.46, 95% CI 1.77–23.65, *p* = 0.003) (Table 2). On multivariate analysis, upper/middle lobe lung lesion location was no longer significantly associated with chest drain insertion (adjusted OR 1.81, 95% CI 0.22–15.00, *p* = 0.584).

### Presence of emphysema

Chest drain insertion rates following pneumothorax aspiration was significantly higher following biopsy of lesions in patients with emphysema (29.4%) compared to those without emphysema (11.8%) (OR 3.13, 95% CI 1.10–8.87, *p* = 0.028) (Table 2). On multivariate analysis, the presence of emphysema was no longer significantly associated with chest drain insertion (adjusted OR 2.51, 95% CI 0.58–10.90, *p* = 0.219).

### Longer needle insertion depth

Chest drain insertion rates following pneumothorax aspiration was significantly higher for a longer biopsy needle depth (from parietal pleural to tip of biopsy needle), with a mean of 34.7 mm in patients that required chest drain insertion and 21.0 mm for those successfully treated with aspiration (*p* = 0.005) (Table 4). The chest drain insertion rate was 50% for a needle depth ≥5 cm, 37.5% for ≥4 cm, 36.7% for ≥3 cm, 34.7% for ≥2 cm, 25.0% for ≥1 cm and 7.7% for <1 cm (Table 2). Chest drain insertion rates was almost four times more likely if the pneumothorax biopsy needle depth was ≥2 cm compared to <2 cm (OR 4.00, 95% CI 1.44–11.07, *p* = 0.005). The univariate analyses for different biopsy needle depth cut-offs is summarized in Table 2.

**Table 4.** Comparison of continuous characteristics between patients with pneumothoraxes after CT-PTLB who were successfully aspirated compared to those who required chest drain insertion

Characteristic	PTX treated with aspiration only (*n* = 81)	PTX required chest drain insertion after aspiration (*n* = 21)	*p-value^a^*
Lesion size (mm)	29.4	26.0	*0.476*
Needle distance (mm)	21.0	34.7	***0.005***
Longest PTX radial depth (mm)	20.8	40.6	***<0.001***

PTLB, percutaneous transthoracic lung biopsy ; PTX, pneumothorax.

a *p-value* derived from independent *t*-test for continuous variables

On multivariate analysis, pneumothorax biopsy needle depth was not significantly associated with chest drain insertion following aspiration (≥2 cm *vs* <2 cm, adjusted OR 3.75, 95% CI 0.86–16.38, *p* = 0.079) (Table 3).

### Factors not associated with chest drain insertion following pneumothorax aspiration

Chest drain insertion following pneumothorax aspiration was not associated with the patient’s sex, age, use of conscious sedation, prior chemotherapy, or recent anticoagulant use. Chest drain insertion rates following pneumothorax aspiration was lower in patients with prior radiotherapy to the thorax (7.1%) compared to those without prior radiotherapy (25.7%), however, this did not reach statistical significance (OR 0.22, 95% CI 0.05–1.03, *p* = 0.053; adjusted OR 0.39, 95% CI 0.06–2.46, *p* = 0.315).

Lung lesion factors not associated with chest drain insertion included lesion composition (subsolid or solid), whether the lesion abutted a fissure, whether the lesion crossed a fissure and whether the lesion had internal cavitation. Biopsy technique factors not associated with chest drain insertion included needle technique (non-coaxial *vs* coaxial), size of biopsy needle gauge, biopsy needle type (Biopince *vs* Temno), biopsy procedure time, number of pleural punctures and CT technique (Helical *vs* Fluoroscopy). An adequate sample for pathology analysis was also not associated with chest drain insertion.

### Complication and mortality rate

No significant hemorrhage, air embolism or mortality occurred during or after the aspiration. No complications associated with chest drain insertion were reported.

## Discussion

This study demonstrated the two key factors which have the greatest impact on whether or not pneumothorax aspiration will be successful in avoiding chest drain insertion are pneumothorax size and biopsy position. With respect to pneumothorax size, our results are concordant with Yamagami and colleagues.^6^ Their larger cut-off >543 ml of aspirated air being successful in almost half of aspiration attempts is similar to this current study of radial pneumothorax depth cut-offs at ≥3 cm or ≥4 cm having a 50% success rate. Although some proceduralists may take the perspective that aspiration should not be attempted in patients with a larger pneumothorax, we view these findings in the alternative light, whereby aspiration of a large pneumothorax can avoid chest drain insertion in around 50% of patients.

Another strong independent factor associated with higher rates of chest drain insertion following pneumothorax aspiration was a supine biopsy position. We hypothesize one of the main reasons for these findings is due to the “roll-over effect” whereby patients who were biopsied/aspirated in the prone position were placed in a supine position afterwards and thus rolled-over. Any hemorrhage created from biopsy would be dependent and contribute to occluding the needle tract and reducing the likelihood of the already established pneumothorax from enlarging. A previous meta-analysis demonstrated a pooled threefold reduction in the risk of chest drain insertion when the patient was rolled over to puncture site down immediately (8.6% *vs*  24.4%, pooled OR 0.34, 95% CI 0.18–0.63, *p* < 0.001).^4,12,13^ These findings suggest the risk of pneumothorax enlarging following biopsy and/or aspiration may be reduced if patients are placed with the puncture site down following biopsy/aspiration.

This study also identified several other factors associated with increased chest drain insertion following aspiration, such as upper/middle lobe location, presence of emphysema and longer needle insertion depth, however, these were no longer statistically significant after controlling for pneumothorax depth and supine position on multivariate analysis. Furthermore, coaxial technique had a lower chest drain insertion rate compared to the non-coaxial technique (18.2% *vs*  28%), however, this was not statistically significantly different (OR 0.57, 95% CI 0.20–1.62, *p* = 0.291). Future research assessing the effectiveness of different aspiration techniques on chest drain insertion risk would be useful.

This study had several limitations. Firstly, the method of measuring pneumothorax size is far from precise compared to measured aspirated volumes. The geometry of the thorax also indicates that linear increases in radial depth have at least an r^2^ to r^3^ relationship to the pneumothorax volume. The provided longest direct lung to parietal pleura is at best a rough estimation, but one that is easily attainable and measured at the end of a CT-PTLB. Secondly, this study is retrospective, containing inherent biases in design and data acquisition. Thirdly, the operators and rationale for aspiration were heterogeneous. Over a 10-year period, there have been multiple proceduralists who have different interpretations of departmental guidelines for attempting aspiration. Lastly, few patients who lived out of area and may have required a drain at an external health service post-biopsy and their complication rate may not be accounted for.

Future research in this area is warranted given the relative paucity of data but potential significant benefit for patients and reduction in hospitalization costs. With improvement in technology and iterative research leading to the identification of risk factors and post-biopsy manoeuvres leading to reduced complications, larger cohorts are required to see small differences in rare outcomes. Larger studies may elicit other relationships that can lead to a stronger correlation with successful or unsuccessful outcomes. If prospective studies are conducted, consideration of a volumetric ultra-low dose technique to acquire the entire thorax to accurately derive pneumothorax volumes could be useful.

## Conclusion

Aspiration of pneumothorax after CT-guided lung biopsy is a straightforward addition for reducing further complications. Although larger pneumothoraxes have higher risks of aspiration failure requiring a chest drain, around half of these attempts are successful and thus may be a worthwhile technique to improve patient outcomes, reduce chest drain insertion and hospitalization costs.
