# Supplementary material for: Manual aspiration of a pneumothorax after CT-guided lung biopsy: outcomes and risk factors
Source: Br J Radiol. 2023 Jun 28;96(1148):20220366. doi: 10.1259/bjr.20220366 (PMC10392636; doi:10.1259/bjr.20220366)
Supplement: Supplementary Table 1. [file bjr.20220366.suppl-01.docx]

**SUPPLEMENTARY TABLE**

**Supplementary Table 1: Risks of pneumothorax with adjusted groups.**

|  | **Chest Drain Insertion after Pneumothorax Aspiration** | | **OR**  **(95%CI)** |
| --- | --- | --- | --- |
|  | **Group A** | **Group B** |  |
| Longest Radial Pneumothorax Depth |  |  |  |
| 1-2 cm [A] vs ≥ 2 cm [B] | 0% | 36.8% | N/A |
| 1-3cm [A] vs ≥ 3 cm [B] | 7.1% | 50.0% | 13.00 (3.81 – 44.00)*** |
| 1-4cm [A] vs ≥ 4 cm [B] | 13.6% | 50.0% | 6.33 (2.18-18.37)*** |
| 2-3cm [A] vs ≥ 3 cm [B] | 17.4% | 50.0% | 4.75 (1.33 – 16.93)** |
| 3-4cm [A] vs ≥ 4 cm [B] | 35.7% | 50.0% | 1.80 (0.46 – 6.97) |

*P-value <0.05; **P-value<0.01, ***p-value <0.001; 95%CI - 95% Confidence Interval; OR, Odds Ratio; N/A, Not Applicable
